# Comparative Efficacy and Safety of Treatments for Parkinson's Disease With Depression: A Systematic Review and Bayesian Model‐Based Network Meta‐Analysis

**DOI:** 10.1002/brb3.71722

**Published:** 2026-08-25

**Authors:** Yuhang Zhao, Qing'er Mo, Yesong Liu, Xiaohan Chen, Qianhui Zhao, Huiqian Wang, Zhenzhen Yan, Lanxiao Cao, Guohua Zhao

**Affiliations:** ^1^ Department of Neurology, the Fourth Affiliated Hospital of School of Medicine, and International School of Medicine International Institutes of Medicine Zhejiang University Zhejiang China

**Keywords:** depression, network meta‐analysis, Parkinson's disease, treatments

## Abstract

**Objective:**

Depression is a common non‐motor symptom of Parkinson's disease (PD) and substantially impairs patients’ quality of life. This study aimed to compare the efficacy and safety of different interventions for depression in PD.

**Methods:**

We conducted a systematic review and Bayesian network meta‐analysis of randomized controlled trials (RCTs) in patients with PD and depression. Databases were searched from inception to September 30, 2025. Treatment effects were estimated using standardized mean differences (SMDs) for efficacy and odds ratios (ORs) for safety. Surface Under the Cumulative Ranking Curve (SUCRA) values were used to rank interventions. The protocol was registered in PROSPERO (CRD420251245403).

**Results:**

This study included 51 RCTs involving 5100 patients and 42 intervention measures. Compared with placebo, citalopram (SMD = −0.99, 95% CI: −1.87 to −0.12) and primary motor cortex (M1) repetitive transcranial magnetic stimulation (rTMS) (SMD = −1.29, 95% CI: −1.93 to −0.64) significantly improved depressive symptoms with moderate‐quality evidence. SUCRA rankings favored citalopram (0.831), M1 rTMS (0.825), pergolide (0.811), and supplementary motor area (SMA) + M1 rTMS (0.802), although rankings should be interpreted alongside direct statistical comparisons. Several treatments could not be included in the safety network because of insufficient connectivity. Citalopram, sertraline, rasagiline, methylphenidate, memantine, and trazodone were associated with higher withdrawal rates due to adverse events.

**Conclusion:**

Our findings indicate that selective serotonin reuptake inhibitors (SSRIs), dopamine agonists, and rTMS suggest relative efficacy and an acceptable safety profile in treating PD with depression. However, given the limited evidence for several interventions, these findings should be interpreted cautiously and confirmed in larger, multicenter studies.

## Graphical abstract

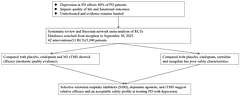

## 1 Introduction

Parkinson's disease (PD) is a progressive neurodegenerative disorder characterized not only by motor symptoms such as tremor, bradykinesia, and rigidity but also by a range of non‐motor symptoms that significantly impair patients’ quality of life (Morris et al. 2024). Among these, depression stands as one of the most prevalent and debilitating neuropsychiatric comorbidities, affecting approximately 40% of PD patients (Ahmad et al. 2023). Depressive symptoms in PD patients are associated with accelerated cognitive decline, more severe functional impairment, and reduced treatment adherence, thereby exacerbating the overall burden of the disease (Mou et al. 2022).

The pathophysiological mechanisms underlying PD‐related depression are complex and multifactorial, involving not only psychosocial handicap but also neurobiological alterations associated with dysfunction in dopaminergic, serotonergic, and noradrenergic systems (Galts et al. 2019). Despite its high prevalence and significant clinical importance, PD‐related depression remains underdiagnosed and undertreated in clinical practice. The pharmacological treatment of depression in PD has primarily focused on selective serotonin reuptake inhibitors (SSRIs) and tricyclic antidepressants (TCAs) (Assogna et al. 2020). Additionally, behavioral interventions (such as psychotherapy), electroconvulsive therapy, repetitive transcranial magnetic stimulation (rTMS), and deep brain stimulation (DBS) have also demonstrated efficacy in treating PD depression (Laux 2022). A previous network meta‐analysis (NMA) demonstrated that dopamine agonists (DOP) and SSRIs show good efficacy in PD depression (Wang et al. 2022). However, this previous analysis had several important limitations. It excluded non‐pharmacological interventions, such as rTMS and fecal microbiota transplantation (FMT), and its literature search was completed before 2022, thereby missing several recently published randomized controlled trials (RCTs). In addition, the scope of evaluated interventions was relatively limited, comprising fewer than 20 treatment options. To address these gaps, the present study systematically integrates both pharmacological and non‐pharmacological interventions into a single network encompassing 42 treatment strategies, updates the evidence base through September 2025, applies the latest Risk of Bias tool 2.0 (RoB 2.0) and CINeMA frameworks to assess risk of bias and certainty of evidence, and provides a comprehensive evaluation of safety across two distinct safety outcomes.

Traditional RCTs are typically designed to compare a single intervention with a placebo or another intervention; however, when multiple treatment options exist, conducting RCTs that directly compare them pairwise is often costly or difficult to implement, leading to a lack of head‐to‐head evidence. NMA offers an effective strategy for integrating all available RCT data to comprehensively evaluate the relative efficacy and safety of multiple treatment options. Given the recent increase in RCTs on PD depression treatment, it is crucial to identify which treatment options achieve the optimal balance between efficacy and tolerability. Although their mechanisms of action differ, both medication and rTMS are commonly used clinical interventions for treating depression in PD. To provide clinicians with a comprehensive comparison of the relative efficacy and safety of all available treatment strategies, we included both pharmacological and non‐pharmacological interventions in this NMA. This study aims to compare the efficacy and safety of various treatments for PD depression through a systematic review and NMA of RCTs. By synthesizing existing evidence, we expect to provide an updated, evidence‐based ranking of antidepressant interventions to guide clinicians in optimizing treatment strategies.

## 2 Methods

This review was conducted in full compliance with the PRISMA‐NMA reporting guidelines. To enhance transparency and reproducibility, the study protocol was prospectively registered with the PROSPERO international registry of systematic reviews (CRD420251245403). The analysis method was adapted from a prior NMA (J. Zhang et al. 2024). The present study specifically focuses on short‐ to medium‐term treatment outcomes, generally defined as endpoint assessments conducted within a follow‐up period of 4–24 weeks, consistent with the available RCT evidence base. Long‐term efficacy and safety (beyond 6 months) remain an important unaddressed gap, as discussed in the Limitations section.

### 2.1 Literature Search and Study Selection

A systematic search of electronic databases was performed from database inception through September 30, 2025. The following sources were searched without language restrictions: MEDLINE, Web of Science (WOS), Embase, Scopus, and the Cochrane Central Register of Controlled Trials. A combination of controlled vocabulary and keyword terms was applied to capture studies related to “PD,” “depression,” “RCTs,” and various interventions. Table S1 presents the complete search strategy. Additionally, the reference lists of relevant review articles were manually screened to identify any additional eligible studies. Eligible studies were required to enroll patients meeting internationally recognized criteria for idiopathic PD. Depression was defined by a validated rating scale score meeting pre‐specified threshold criteria as reported in each individual trial or by a formal clinical diagnosis documented by the original study authors. No restrictions were imposed on PD disease duration, patient age, or background anti‐parkinsonian medications in order to maximize the breadth of evidence synthesis. However, we acknowledge that this inclusiveness introduces clinical heterogeneity, which is discussed as a limitation.

Double‐blind RCTs were selected for this review. This design was chosen to increase methodological soundness and decrease the likelihood of bias. Study population: Patients with primary PD meeting internationally recognized diagnostic criteria (Postuma et al. 2018), regardless of depression severity or prior antidepressant treatment. Interventions: Any treatment modality for depression in PD. Controls: Placebo or other active medications. Primary Outcomes: Change in depression symptom scores (Beck Depression Inventory [BDI], Hamilton Depression Rating Scale [HAMD], Montgomery‐Asberg Depression Rating Scale [MADRS], Geriatric Depression Scale [GDS], etc.). Secondary outcomes: All‐cause discontinuation rate and adverse event incidence. In this study, safety was assessed using two measures: (a) the overall incidence of adverse events (the overall proportion of any adverse events); and (b) the withdrawal rate due to adverse events. Exclusion criteria: non‐RCT studies, studies lacking relevant outcome data, and studies with non‐extractable data. If multiple studies reported identical or overlapping patient data, the most recent and comprehensive study of the patient cohort was included.

Two researchers (Yuhang Zhao and Qing'er Mo) independently extracted the following data: (A) Study basic information: authors, publication year, sample size. (B) Subject characteristics: age, gender, disease duration. Intervention details: intervention measures, type and value of outcome measures. Outcome data: depression scale scores, number of responders, number of adverse events.

### 2.2 Outcome Measures and Data Synthesis

To handle variability in depression rating instruments (such as BDI, HAMD, and MADRS) across trials, the standardized mean difference (SMD) was chosen as the effect size metric for changes in depressive symptom scores. For studies reporting only baseline and post‐treatment means with standard deviations, the pooled standard deviation for pre‐to‐post changes was estimated following the procedure outlined in Cochrane Handbook (version 5.1.0, Chapter 16.1.3.2). Dichotomous outcomes were expressed as odds ratios (ORs) with 95% confidence intervals (CIs).

All analyses were conducted using random‐effects models to account for between‐study heterogeneity. For the NMA, SMDs expressed as Hedges’ g were used when outcomes were measured using different instruments, thereby allowing effects to be compared across scales while correcting for small‐sample bias. For pairwise meta‐analyses in which studies used the same outcome scale, mean differences in change from baseline were calculated directly from the reported means, standard deviations, and sample sizes to retain the original measurement unit and facilitate clinical interpretation. Direct evidence was first synthesized using pairwise meta‐analysis. Statistical heterogeneity was assessed using Cochran's *Q* test and quantified with the *I* ^2^ statistic, with values of ≤ 25%, 25%–75%, and ≥ 75% interpreted as low, moderate, and high heterogeneity, respectively. Because only a limited number of studies contributed to each direct comparison, no subgroup analyses were prespecified. Publication bias was evaluated using funnel plots and Egger's regression test when a sufficient number of studies were available.

A Bayesian hierarchical framework was used for the NMA, implemented with the GEMTC package (version 1.0‐2) in R (version 4.3.2). The analysis ran on a Bayesian Markov chain Monte Carlo (MCMC) algorithm via the JAGS (Just Another Gibbs Sampler) engine. Four independent chains were run, each with 20,000 adaptation iterations followed by 50,000 simulation iterations to guarantee sufficient exploration of the parameter space. Model fit was evaluated using trajectory density plots and convergence diagnostics. A potential scale reduction factor (PSRF) close to 1 was considered evidence of satisfactory convergence; otherwise, further iterations were performed.

Treatment rankings were based on the Surface Under the Cumulative Ranking Curve (SUCRA), expressed as a percentage—higher values indicate better performance. Consistency between direct and indirect evidence was assessed using node‐splitting. Adjusted funnel plot asymmetry tests were also performed to detect possible publication bias and small‐study effects.

### 2.3 Risk of Bias and Certainty Assessment

Two assessors (Yuhang Zhao and Qing'er Mo) independently assessed the risk of bias for each included RCT using the revised Cochrane RoB 2.0. Evidence quality was graded using the GRADE approach combined with the CINeMA tool, assigning NMA results to one of four levels: high, moderate, low, or very low. Assessment dimensions included risk of bias within studies, indirectness, inconsistency, imprecision, publication bias, and dose‐response relationships. Discrepancies between the two reviewers were resolved through structured discussion; when consensus could not be reached, a third senior investigator (Guohua Zhao) served as the arbiter.

## 3 Results

### 3.1 Selection of Studies and Bias Risk Analysis

**FIGURE 1:**
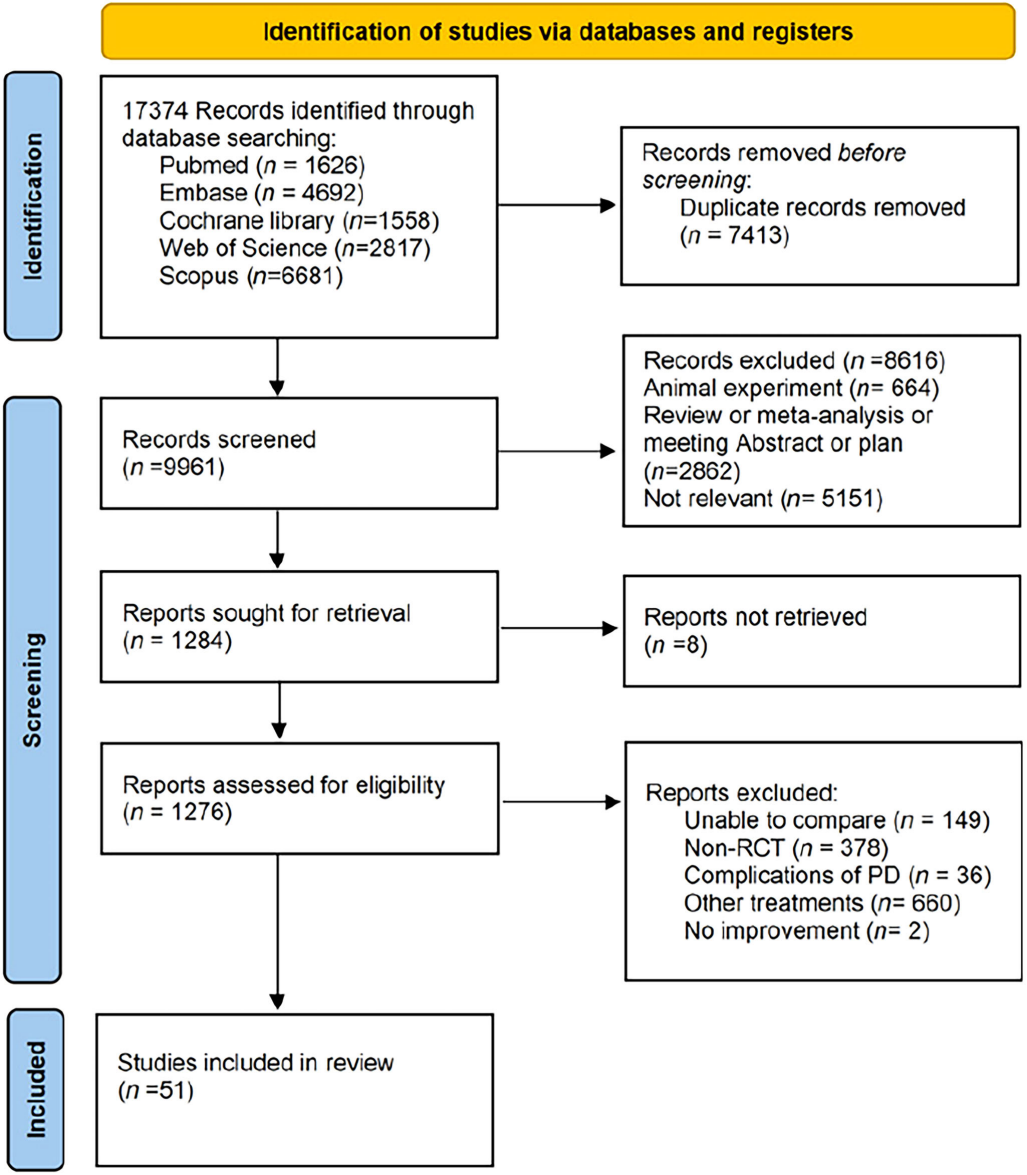
Flowchart of study selection according to PRISMA guideline.

We searched the included databases and retrieved 17,374 records. After removing duplicates, 9961 abstracts were screened, and 90 full‐text articles underwent thorough review. Among these studies, 51 studies (Avila et al. 2003; Allain et al. 1991; Makkos et al. 2016; Cattaneo et al. 2017; Flohr and Breull 1975; Khedr et al. 2024; Pal et al. 2010; Ziaei et al. 2022; Richard et al. 2012; Chen et al. 2022; Rektorová et al. 2003; Aftanas et al. 2021; Meloni et al. 2020; Pomponi et al. 2014; Serrano‐Dueñas 2002; Menza et al. 2009; Rascol et al. 2012; Barone et al. 2006; Boggio et al. 2005; Barone et al. 2010; Daneshvar Kakhaki et al. 2020; Pahwa et al. 2007; Jiang et al. 2023; Mehrabani et al. 2023; Thobois et al. 2013; Group 1993; Song et al. 2024; Zhu et al. 2025; Z. Zhang et al. 2025, 2013; Ardenne and Reitnauer 1975; Benninger et al. 2010; Brys et al. 2016; Castrioto et al. 2020; Cheng et al. 2023; Chung et al. 2016; Espay et al. 2011; Hanagasi et al. 2011; Kanjanarangsichai et al. 2022; Athauda et al. 2017; Ondo et al. 2005; Shirota et al. 2013; Parkinson Study Group 2002; Tsang et al. 2000; Wermuth et al. 1998; Mitarnun et al. 2025; Magistrelli et al. 2024; Hauser et al. 2016; Werneck et al. 2009; Trenkwalder et al. 2011; Ondo et al. 2011) met our inclusion criteria, as shown in Figure 1. All trials adopted a parallel‐group design. Disease duration across studies ranged from 2 to 9.7 years. A variety of instruments were used to assess primary outcomes, including the BDI, HAMD, MADRS, and GDS. Detailed baseline and design features of the included studies are summarized in Table S2. In total, 5100 patients were analyzed across 42 treatment strategies, comprising 33 pharmacological agents and 9 non‐drug interventions—specifically rTMS and FMT.

**FIGURE 2:**
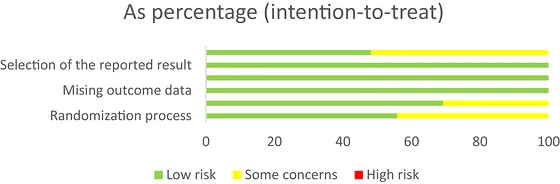
Risk of bias plot: RCTs.

The Cochrane Risk of Bias Tool was applied to evaluate potential bias in all eligible RCTs. Based on the assessment, 25 studies were classified as having “some concerns,” mainly due to insufficient specification of the randomization procedure. The remaining 26 studies received a judgment of “low risk of bias.” An overview of the risk of bias is displayed in Figure 2, while detailed evaluations for each study are available in Figure S1. To evaluate the potential influence of studies rated as having “some concerns” for risk of bias, we performed sensitivity analyses by stratifying the network according to risk‐of‐bias category (low risk vs. others) and comparing the pooled effect estimates and treatment rankings (SUCRA). The results remained broadly consistent with the primary analysis, with overlapping CIs and no meaningful changes in treatment rankings. For the two interventions showing the most favorable effects in the primary analysis, citalopram (SMD = −1.19, 95% CI: −2.75 to 0.37) and M1 rTMS (SMD = −0.98, 95% CI: −1.76 to −0.20), the effect estimates were generally preserved, suggesting that the inclusion of studies with “some concerns” did not materially influence the overall findings. Accordingly, the certainty of evidence was downgraded by one level for risk of bias in the GRADE assessment, resulting in moderate‐certainty evidence rather than indicating serious concerns about the robustness of the findings.

### 3.2 Pairwise Meta‐Analysis

Pairwise meta‐analyses were performed for several interventions, including dorsolateral prefrontal cortex (DLPFC) rTMS (three trials), primary motor cortex (M1) rTMS (four trials), rasagiline (three trials), and rotigotine (four trials). Compared with placebo, two interventions showed statistically superior antidepressant effects: M1 rTMS (SMD = −0.92, 95% CI: −1.49 to −0.36; *I* ^2^ = 64.3%) and rotigotine (SMD = −0.19, 95% CI: −0.35 to −0.03; *I* ^2^ = 0%). In contrast, DLPFC rTMS did not differ significantly from placebo in terms of efficacy. Safety outcomes were examined for rTMS (5 studies) and rotigotine (4 studies). rTMS was associated with a favorable safety profile (OR = 3.51, 95% CI: 0.88–13.99; *I* ^2^ = 0%), whereas rotigotine showed an elevated adverse event rate relative to placebo (OR = 1.14, 95% CI: 1.04–1.26; *I* ^2^ = 0%). For rotigotine, the rate of treatment discontinuation due to adverse events was not significantly different from that of placebo (Figures S2–S9). For rTMS interventions, we did not impose restrictions on stimulation parameters (frequency: high‐frequency > 1 Hz vs. low‐frequency ≤ 1 Hz; intensity: percentage of resting motor threshold; number of pulses per session; and total number of sessions) as a priori eligibility criteria. This decision was made to maximize the available evidence base, but it inevitably introduces technical heterogeneity. A descriptive summary of rTMS parameters for all included rTMS trials is provided in Table S3. Of note, the high heterogeneity observed in rTMS pairwise analyses (*I* ^2^ = 86.3%) is likely partly attributable to these parameter differences. Therefore, the above findings warrant cautious interpretation.

### 3.3 NMA of All Treatments

**FIGURE 3:**
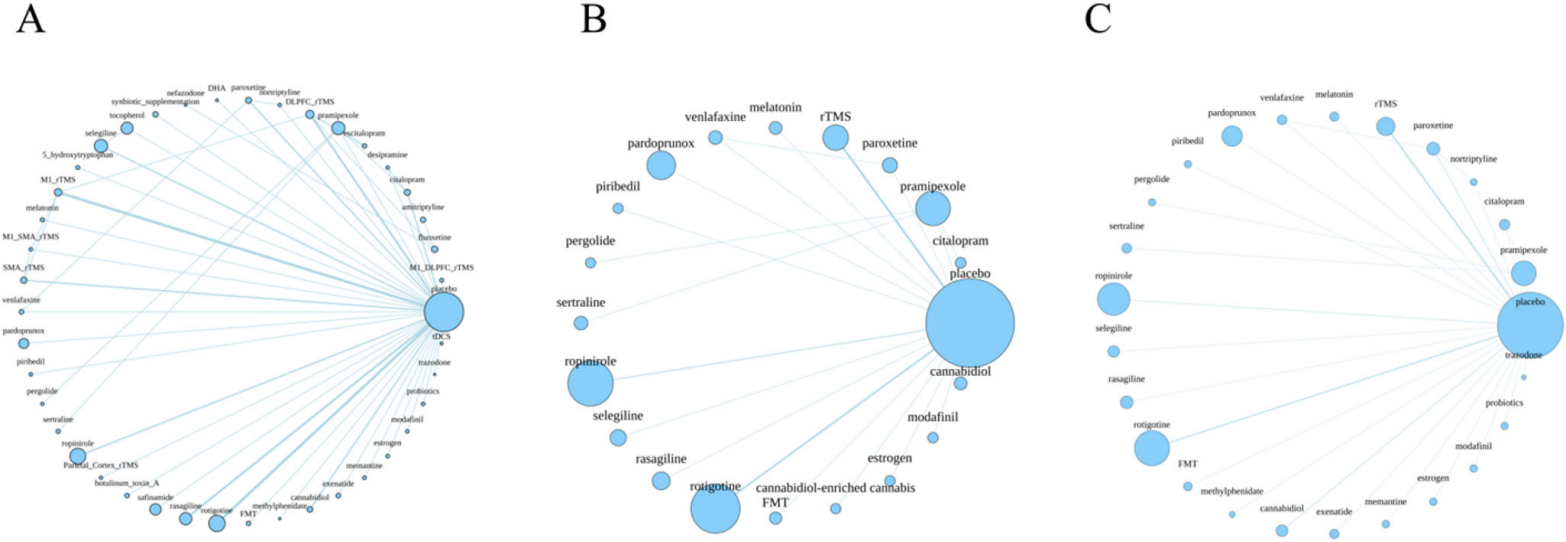
Network diagram. (A) for comparing all treatments efficacy. (B) for comparing all treatments safety. (C) comparing all treatments adverse reaction withdrawal rate. rTMS, fecal microbiota transplantation (FMT), and transcranial direct current stimulation (tDCS).

Data from all 51 RCTs were synthesized in an NMA (Figure 3). Relative to placebo, five interventions showed statistically significant antidepressant effects: citalopram (SMD = −0.99, 95% CI: −1.87 to −0.12), escitalopram (SMD = −1.38, 95% CI: −2.7 to −0.05), nortriptyline (SMD = −1.30, 95% CI: −2.57 to −0.03), M1+SMA rTMS (SMD = −1.32, 95% CI: −2.49 to −0.14), and M1 rTMS (SMD = −1.29, 95% CI: −1.93 to −0.64). We further assessed treatment safety by analyzing the number of participants experiencing adverse events and study withdrawals due to adverse events. The NMA included 31 studies on the incidence of adverse events. Results indicated that pardoprunox (OR = 9.6, 95% CI: [3.2, 32]) and rTMS (OR = 10.7, 95% CI: [1.7,232]) carried a significantly higher risk of adverse events. Pardoprunox‐associated adverse events primarily included nausea, dizziness, somnolence, and orthostatic hypotension, as reported in the source trials. rTMS‐related adverse events were generally mild and transient, consisting mainly of scalp discomfort, headache, and dizziness during or immediately after stimulation sessions, consistent with the known tolerability profile of the intervention. The safety NMA for adverse event incidence included 26 studies (*n* = 3168 participants) encompassing 20 interventions, whereas the analysis for withdrawal due to adverse events included 33 studies (*n* = 3440 participants) encompassing 25 interventions. Multiple drugs (sertraline, escitalopram, etc.) were excluded from the safety network, and their exclusion was due to insufficient direct or indirect connections within the network graph, precluding valid indirect inference. Citalopram, sertraline, rasagiline, methylphenidate, memantine, and trazodone resulted in more study withdrawals due to adverse events compared to placebo. No significant differences were observed between placebo and the remaining treatments (Figures S10–12) (Supporting Information 1–3). The comparison‐adjusted funnel plot showed the primary symmetry of studies around the midline (Figure S13). Additionally, two studies positioned directly below and near the funnel plot indicated the presence of small‐sample effects. For pairwise comparisons between statistically significant interventions, the CINeMA tool assessed the certainty of evidence, indicating “moderate” certainty.

### 3.4 Comparative Efficacy: Sucra Rankings

**FIGURE 4:**
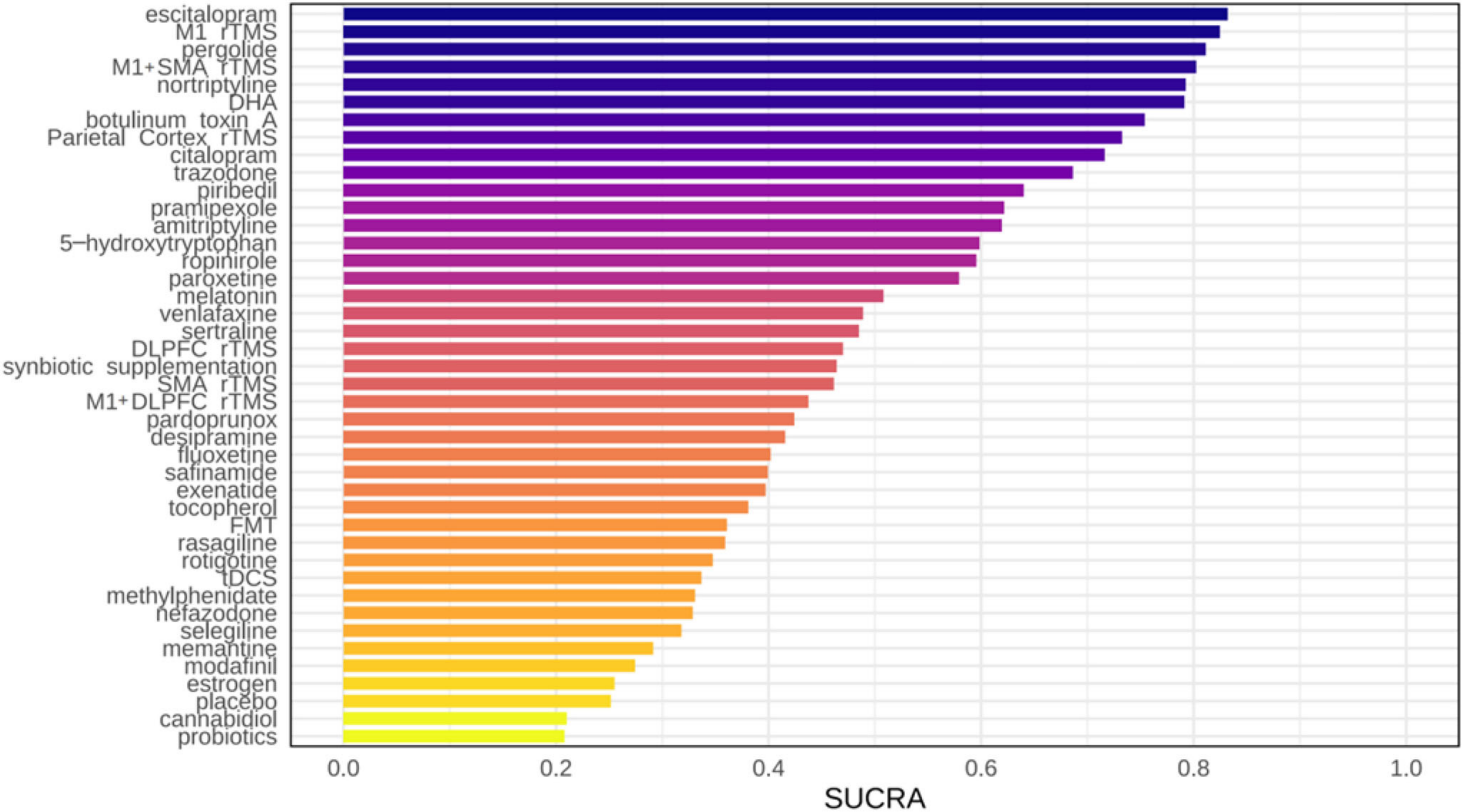
SUCRA ranking of all treatments.

Our study examined SUCRA rankings following treatment. When evaluating treatment efficacy, the SUCRA value rankings were as follows: escitalopram, M1 rTMS, pergolide, SMA + M1 rTMS, nortriptyline, DHA, botulinum toxin A, parietal cortex rTMS, trazodone, citalopram, piribedil, amitriptyline, and pramipexole. Two interventions showed SUCRA values below that of placebo, indicating a lower probability of being beneficial relative to placebo. These included cannabidiol (SUCRA = 0.21, SMD = 0.20, 95% CI: −0.81 to 1.2) and probiotics (SUCRA = 0.15, SMD = 0.23, 95% CI: −1.3 to 1.6) (Figure 4). SUCRA rankings are purely probabilistic and must be interpreted alongside the certainty of evidence, clinical safety profiles, regulatory status, and guideline recommendations before any clinical inference is drawn.

### 3.5 Consistency Assessment and Convergence Diagnostics

Node‐splitting analysis was performed to examine potential discrepancies between direct and indirect effect estimates. After applying this approach, no significant inconsistency or heterogeneity in study quality was detected across the network. These findings support the use of the consistency model as an appropriate and dependable framework for our analysis. The PSRF was maintained below 1 for all parameters, indicating that the Markov chains achieved satisfactory convergence. This confirms the stability and reliability of the estimated results.

## 4 Discussion

This NMA integrated both direct and indirect comparisons from 51 RCTs, encompassing 5100 PD patients and 42 distinct interventions. The primary objective was to compare the relative efficacy and safety profiles of available treatment strategies for PD‐related depression. Furthermore, a probabilistic ranking of interventions was generated to help identify potentially optimal therapeutic options. NMA results demonstrated that citalopram and M1 rTMS outperformed placebo in alleviating PD depression symptoms. Additionally, according to SCURA, treatments such as escitalopram, sertraline, and pramipexole demonstrated marked symptom improvement compared to placebo. Among all treatments, rTMS, SSRI, and dopamine agonist (DOP) ranked highest in relative efficacy. Regarding safety assessments, based on NMA, we observed higher adverse reaction rates for SSRIs and rTMS compared to placebo. According to the NMA, most interventions did not differ significantly from placebo in terms of adverse event risk. Similarly, the remaining treatments showed comparable safety profiles to placebo, with no statistically significant elevations in adverse reaction rates.

Depression frequently emerges as a prodromal feature of PD, yet its role in disease pathogenesis remains widely underappreciated. A growing body of experimental evidence suggests that depression may play a dual role, acting both as a cause and a consequence of PD. On the one hand, depression may be a direct consequence of α‐synuclein pathology spreading to limbic and brainstem regions critical for emotional regulation, including the nucleus accumbens, locus coeruleus, and hippocampus (Yusuf et al. 2025). Conversely, chronic psychological stress combined with dysregulation of the hypothalamic‐pituitary‐adrenal (HPA) axis may promote α‐synuclein aggregation. Taken together, these observations suggest that depression, as a non‐motor feature of PD, contributes to disease pathogenesis and may accelerate its worsening or progression across different phases (Yusuf et al. 2025). This suggests that the treatment for depression in PD is not only about symptom control but may also have a potential impact on the disease progression. Therefore, it is particularly important to systematically evaluate the benefit‐risk ratio of various treatment strategies.

For a long time, evidence regarding the tolerability, safety, and efficacy of antidepressants in patients with PD has remained limited. The most extensively studied medications for depression in PD are SSRIs and TCAs (Bang et al. 2021), whereas serotonin‐norepinephrine reuptake inhibitors (SNRIs), DOP, trazodone, mirtazapine, and atomoxetine have been investigated less extensively. To provide a more up‐to‐date clinical context, we added the 2024 clinical review by Tanner and Ostrem (2024), the Brazilian Academy of Neurology consensus on non‐motor symptoms in PD (Maia et al. 2025), and the German Society for Neurology guideline on the diagnosis and treatment of PD (Höglinger et al. 2024). These publications generally recommend an individualized and multimodal treatment strategy, including optimization of dopaminergic therapy and appropriate use of antidepressants such as SSRIs, SNRIs, selected TCAs, and DOP, as well as non‐pharmacological interventions such as cognitive behavioral therapy and physical exercise. Treatment selection should be tailored to depression severity, predominant symptoms, comorbidities, potential drug interactions, and individual tolerability.

### 4.1 DOP

This NMA suggests that DOP, including pergolide, pramipexole, and ropinirole, may be effective in alleviating depressive symptoms in patients with PD, consistent with evidence supporting the role of dopaminergic therapy in managing depression in this population and with recommendations from several clinical guidelines. However, an important interpretive caveat should be emphasized. Although pergolide ranked third in the SUCRA analysis, this ranking reflects only its statistical probability of being among the more efficacious interventions within the analyzed evidence network and should not be interpreted as a recommendation for clinical use. Pergolide is an ergot‐derived dopamine agonist that has been withdrawn from most markets worldwide and is no longer recommended by major clinical guidelines because of its well‐established association with serious cardiac valvulopathy, including aortic and mitral valve regurgitation (Jing et al. 2023). Its relatively favorable ranking should therefore be interpreted cautiously, particularly given the limited evidence base and the uncertainty inherent in probability‐based ranking metrics. Importantly, SUCRA rankings reflect relative probabilities rather than overall clinical value and should always be considered alongside treatment safety, real‐world availability, and current guideline recommendations. In contrast, non‐ergot DOP such as pramipexole and ropinirole also demonstrated beneficial effects in this NMA while maintaining more favorable safety profiles. Consistent with these findings, the Movement Disorder Society evidence‐based review recommends pramipexole as an effective treatment option for PD patients with comorbid depression (Seppi et al. 2019).

### 4.2 SSRI

Our analysis supports the efficacy of SSRIs such as citalopram and sertraline. Escitalopram, a selective SSRI, has demonstrated efficacy and tolerability in patients with depression beyond PD (Cipriani et al. 2018). Previous studies on sertraline, paroxetine, and fluoxetine for PD showed minimal efficacy differences, with insufficient evidence to support their use for treating PD (Seppi et al. 2011), potentially due to inconsistent levels of research evidence. In this NMA, citalopram demonstrated significant antidepressant effects, strongly supporting the positive role of SSRIs in treating PD‐related depression. Additionally, other SSRIs, such as sertraline and paroxetine, demonstrated efficacy in this NMA. However, this conclusion differs from those of some previous pairwise meta‐analyses on PD, which failed to show significant superiority over placebo. This discrepancy may be attributed to the incorporation of a broader spectrum of indirect comparison evidence via NMA, or it may reflect methodological heterogeneity across prior studies. It should be noted that when SSRIs are used in elderly patients with PD, attention should be paid to drug interactions and the potential risk of cardiotoxicity. Therefore, “professional monitoring” combined with regular electrocardiogram (ECG) monitoring is recommended (Galts et al. 2019).

### 4.3 Other Drugs

Nortriptyline demonstrated superiority over placebo in reducing HDRS‐17 scores (*p* = 0.002, effect size 1.20), whereas paroxetine CR did not show a statistically significant difference (*p* = 0.165, effect size 0.51) in a double‐blind, randomized, placebo‐controlled study by Menza et al. (2009). Despite the limited sample size, nortriptyline remains likely efficacious for treating depression in PD. Following Hibbeln's initial report (Hibbeln 1998), epidemiological studies have frequently linked symptoms of depression to variations in the intake of n‐3 fatty acids, specifically EPA and DHA. This observed association may be attributed to the potential anti‐inflammatory properties of these fatty acids. DHA and EPA are not only cost‐effective and easily integrated into clinical practice, but they may also serve as a neuroprotective or disease‐modifying approach to potentially delay the onset of depressive symptoms. It is worth noting that although DHA ranks highly in the SUCRA rankings, DHA achieved a relatively favorable SUCRA ranking (0.791, sixth overall), although this finding was derived from a single small pilot trial and should therefore be regarded as hypothesis‐generating. Future research should prioritize adequately powered RCTs incorporating biomarker‐based assessments to clarify the therapeutic role and mechanisms of DHA in PD‐related depression. Similarly, although FMT represents a promising gut–brain axis–targeted intervention, evidence remains limited to a single randomized trial. Future studies should focus on protocol standardization, mechanistic validation, and multicenter clinical trials to establish its efficacy and safety.

### 4.4 rTMS

rTMS is a non‐invasive brain stimulation method that operates on the principle of electromagnetic induction. It delivers pulsed magnetic fields to the scalp, which generate small electrical currents in targeted cortical regions (Yin et al. 2023). Stimulation frequency determines the classification of rTMS: low‐frequency (≤ 1 Hz, LF‐rTMS) and high‐frequency (> 1 Hz, HF‐rTMS) protocols (Xia et al. 2022). rTMS applied to the prefrontal cortex generates magnetic fields that depolarize subcortical neurons and modulate neural circuits associated with emotional regulation and depressive symptoms. Reduced activity in the left DLPFC correlates with depression in PD. Clinically, right‐sided LF‐rTMS is often used to suppress cortical excitability, while left‐sided HF‐rTMS enhances excitability to balance bilateral prefrontal activity. HF‐rTMS improves mood by releasing dopamine, 5‐HT, glutamate, and BDNF, modulating dopaminergic and serotonergic system function, strengthening prefrontal‐limbic system connectivity, and suppressing abnormal default mode network activity (McClintock et al. 2018). We included a recently published NMA, which reported that different rTMS frequencies exert beneficial effects on depressive symptoms in PD, with 10‐Hz rTMS showing the most favorable overall ranking. The same study also noted that only a few participants experienced transient side effects—including dizziness, headache, or scalp numbness—all of which subsided after rest, and all affected individuals were able to complete the study. These observations collectively support both the efficacy and tolerability of rTMS in this patient population (Xia et al. 2025).

Our NMA ranked M1 rTMS (SUCRA = 0.825) and SMA+M1 rTMS (SUCRA = 0.802) as the most promising targets, whereas DLPFC rTMS was not significantly superior to placebo. However, the leave‐one‐out sensitivity analysis suggested that the DLPFC result was influenced by an individual study with a particularly strong placebo response; excluding this study shifted the pooled estimate toward a greater treatment effect. Therefore, the apparent lack of efficacy of DLPFC stimulation should be interpreted cautiously. The potential advantage of M1 and SMA + M1 may reflect the involvement of motor and striatofrontal dopaminergic circuits in PDe‐related depression. The SMA, as a node linking M1 to prefrontal and limbic regions (Narayana et al. 2012), may serve as an integrative target that simultaneously addresses both motor and mood circuits in PD. Nevertheless, conclusions remain limited by the small number of trials, heterogeneous stimulation protocols, and substantial heterogeneity (*I* ^2^ = 86.3%). Larger, standardized head‐to‐head RCTs are needed to determine the optimal stimulation target.

### 4.5 Limitation

This study has several limitations. First, the evidence base remains limited, as most interventions were evaluated in only a single small RCT with short follow‐up durations. Consequently, the reliability of treatment effect estimates is reduced, and the long‐term efficacy and safety of these interventions cannot be established. Second, although SUCRA provides a useful probabilistic ranking of treatments, it should not be interpreted as a direct measure of clinical superiority, particularly for interventions supported by sparse or low‐certainty evidence. Third, substantial clinical and methodological heterogeneity existed across included studies, including differences in diagnostic criteria for depression, patient characteristics, concomitant medications, and depression rating scales. Although SMDs were used to improve comparability, residual heterogeneity may have influenced the estimated treatment effects. Finally, the limited number of high‐quality studies prevented planned subgroup analyses and restricted the generalizability of our findings. Future research should prioritize large, well‐designed RCTs with longer follow‐up periods, incorporate patient‐reported outcomes and biomarkers, and further explore personalized treatment strategies for depression in PD.

### 4.6 Translating Evidence Into Clinical Practice: A Hierarchical Framework

Based on the synthesis of NMA results, GRADE certainty of evidence, and established safety/guideline data, we propose the following provisional hierarchical framework for the pharmacological and neuromodulatory management of PD‐related depression:

#### 4.6.1 Tier 1: First‐Line Options

Citalopram was the only pharmacological treatment significantly superior to placebo with high‐certainty evidence (SMD = −0.99), although ECG monitoring should be considered because of QTc prolongation risk. M1 rTMS also showed high‐certainty efficacy (SMD = −1.93) and may be suitable for patients who cannot tolerate pharmacotherapy.

#### 4.6.2 Tier 2: Second‐Line Options

Pramipexole and rotigotine may be preferred when improvement in both depressive and motor symptoms is desired, but impulse control disorders should be monitored. Nortriptyline is supported by direct evidence but requires caution because of anticholinergic effects.

#### 4.6.3 Tier 3: Investigational Options

DHA, FMT, cannabidiol, and probiotics are supported only by small, single trials and should currently be limited to research settings.

Despite its high SUCRA ranking, pergolide should not be used because of its established risk of valvulopathy. This framework is provisional and should complement existing guidelines and individualized clinical judgment.

## 5 Conclusion

This study suggests that SSRIs, rTMS, and dopamine agonists demonstrate superior efficacy in alleviating depressive symptoms in patients with PD. Notably, our analysis also highlights the potential therapeutic benefits of lipids such as DHA. These results underscore the pressing requirement for further well‐designed RCTs to strengthen and extend the current body of evidence.

## Author Contributions

**Yuhang Zhao**: writing – original draft. **Qing'er Mo**: validation, supervision, methodology, software. **Yesong Liu**: validation, supervision. **Xiaohan Chen**: writing – review and editing, methodology. **Qianhui Zhao**: writing – review and editing, methodology. **Lanxiao Cao**: writing – review and editing, supervision, resources, methodology, conceptualization. **Huiqian Wang**: writing – review and editing, methodology. **Zhenzhen Yan**: conceptualization. **Guohua Zhao**: writing – review and editing, supervision, funding acquisition.

## Funding

This study was supported by the Key Projects of Zhejiang Provincial Natural Science Fund (No. LZ23H090004) and Scientific Research Fund of Zhejiang University (XY2025079).

## Conflicts of Interest

The authors declare no conflicts of interest.

## Supporting information




**Supplementary Material**: brb371722‐sup‐0001‐SuppMat.xlsx

**Supplementary Material**: brb371722‐sup‐0002‐SuppMat.xlsx

**Supplementary Material**: brb371722‐sup‐0003‐SuppMat.xlsx

**Supplementary Material**: brb371722‐sup‐0004‐SuppMat.docx

## Data Availability

The original contributions presented in the study are included in the article/Supporting Information, further inquiries can be directed to the corresponding author.
