# Supplementary material for: Comparative Efficacy and Safety of Treatments for Parkinson's Disease With Depression: A Systematic Review and Bayesian Model‐Based Network Meta‐Analysis
Source: Brain Behav. 2026 Aug 25;16(8):e71722. doi: 10.1002/brb3.71722 (PMC13507520; doi:10.1002/brb3.71722)
Supplement: Supplementary file 1 [file BRB3-16-e71722-s002.docx]

**PubMed**

#1("Parkinson Disease"[Mesh]) OR (Idiopathic Parkinson's Disease[Title/Abstract] OR Lewy Body Parkinson's Disease[Title/Abstract] OR Paralysis Agitans[Title/Abstract] OR Parkinson*[Title/Abstract])

#2("Depression"[Mesh]) OR (Depressive Symptoms[Title/Abstract] OR Emotional Depression[Title/Abstract] OR depress*[Title/Abstract])

#3(random*[Title/Abstract] OR control*[Title/Abstract])

#4(treatment[Title/Abstract] OR intervention[Title/Abstract] OR therapy[Title/Abstract] OR management[Title/Abstract])

#5((tricyclic antidepressants[Title/Abstract] OR TCA[Title/Abstract] OR amitriptyline[Title/Abstract] OR nortriptyline[Title/Abstract] OR desipramine[Title/Abstract] OR doxepin[Title/Abstract] OR clomipramine[Title/Abstract]) OR

(dopamine agonists[Title/Abstract] OR pramipexole[Title/Abstract] OR rotigotine[Title/Abstract] OR pergolide[Title/Abstract] OR memantine[Title/Abstract] OR ropinirole[Title/Abstract] OR piribedil[Title/Abstract]) OR

(selective serotonin reuptake inhibitors[Title/Abstract] OR SSRI[Title/Abstract] OR citalopram[Title/Abstract] OR escitalopram[Title/Abstract] OR fluoxetine[Title/Abstract] OR sertraline[Title/Abstract] OR paroxetine[Title/Abstract] OR fluvoxamine[Title/Abstract]) OR

(serotonin and norepinephrine reuptake inhibitors[Title/Abstract] OR SNRI[Title/Abstract] OR venlafaxine[Title/Abstract] OR desvenlafaxine[Title/Abstract] OR duloxetine[Title/Abstract] OR milnacipran[Title/Abstract]) OR

(serotonin antagonist and reuptake inhibitor[Title/Abstract] OR trazodone[Title/Abstract] OR nefazodone[Title/Abstract]) OR

(norepinephrine reuptake inhibitor[Title/Abstract] OR reboxetine[Title/Abstract] OR viloxazine[Title/Abstract] OR atomoxetine[Title/Abstract]) OR

(noradrenergic and specific serotonergic antidepressant[Title/Abstract] OR NaSSA[Title/Abstract] OR mirtazapine[Title/Abstract] OR mianserin[Title/Abstract]) OR

(Norepinephrine Dopamine Reuptake Inhibitor[Title/Abstract] OR NDRI[Title/Abstract] OR bupropion[Title/Abstract]) OR

(serotonin modulator and stimulator[Title/Abstract] OR vortioxetine[Title/Abstract]) OR

(monoamine oxidase inhibitors[Title/Abstract] OR rasagiline[Title/Abstract] OR selegiline[Title/Abstract] OR moclobemide[Title/Abstract]) OR

(atypical antipsychotic drugs[Title/Abstract] OR quetiapine[Title/Abstract] OR olanzapine[Title/Abstract]) OR

(tianeptine[Title/Abstract] OR agomelatine[Title/Abstract] OR esketamine[Title/Abstract]))

#6 #1 AND #2 AND #3 AND (#4 OR #5)

**Embase**

#1. 'parkinson disease'/exp OR 'parkinson disease' OR 'idiopathic parkinson disease':ti,ab,kw OR 'lewy body parkinson disease':ti,ab,kw OR 'paralysis agitans':ti,ab,kw OR parkinson*:ti,ab,kw

#2. 'depression'/exp OR 'depression' OR 'depressive symptoms':ti,ab,kw OR 'emotional depression':ti,ab,kw OR depress*:ti,ab,kw

#3. random*:ti,ab,kw OR control*:ti,ab,kw

#4. treatment:ti,ab,kw OR intervention:ti,ab,kw OR therapy:ti,ab,kw OR management:ti,ab,kw

#5. 'tricyclic antidepressant':ti,ab,kw OR 'tca':ti,ab,kw OR amitriptyline:ti,ab,kw OR nortriptyline:ti,ab,kw OR desipramine:ti,ab,kw OR doxepin:ti,ab,kw OR clomipramine:ti,ab,kw OR 'dopamine agonist':ti,ab,kw OR pramipexole:ti,ab,kw OR rotigotine:ti,ab,kw OR pergolide:ti,ab,kw OR memantine:ti,ab,kw OR ropinirole:ti,ab,kw OR piribedil:ti,ab,kw OR 'selective serotonin reuptake inhibitor':ti,ab,kw OR 'ssri':ti,ab,kw OR citalopram:ti,ab,kw OR escitalopram:ti,ab,kw OR fluoxetine:ti,ab,kw OR sertraline:ti,ab,kw OR paroxetine:ti,ab,kw OR fluvoxamine:ti,ab,kw OR 'serotonin and norepinephrine reuptake inhibitor':ti,ab,kw OR 'snri':ti,ab,kw OR venlafaxine:ti,ab,kw OR desvenlafaxine:ti,ab,kw OR duloxetine:ti,ab,kw OR milnacipran:ti,ab,kw OR 'serotonin antagonist and reuptake inhibitor':ti,ab,kw OR trazodone:ti,ab,kw OR nefazodone:ti,ab,kw OR 'norepinephrine reuptake inhibitor':ti,ab,kw OR reboxetine:ti,ab,kw OR viloxazine:ti,ab,kw OR atomoxetine:ti,ab,kw OR 'noradrenergic and specific serotonergic antidepressant':ti,ab,kw OR 'nassa':ti,ab,kw OR mirtazapine:ti,ab,kw OR mianserin:ti,ab,kw OR 'norepinephrine dopamine reuptake inhibitor':ti,ab,kw OR 'ndri':ti,ab,kw OR bupropion:ti,ab,kw OR 'serotonin modulator and stimulator':ti,ab,kw OR vortioxetine:ti,ab,kw OR 'monoamine oxidase inhibitor':ti,ab,kw OR rasagiline:ti,ab,kw OR selegiline:ti,ab,kw OR moclobemide:ti,ab,kw OR 'atypical antipsychotic':ti,ab,kw OR quetiapine:ti,ab,kw OR olanzapine:ti,ab,kw OR tianeptine:ti,ab,kw OR agomelatine:ti,ab,kw OR esketamine:ti,ab,kw

#6. #1 AND #2 AND #3 AND (#4 OR #5)

**Cochrane**

#1 (Parkinson Disease):ti,ab,kw OR (Idiopathic Parkinson's Disease):ti,ab,kw OR (Lewy Body Parkinson's Disease):ti,ab,kw OR (Paralysis Agitans):ti,ab,kw OR (Parkinson*):ti,ab,kw

#2 (Depression):ti,ab,kw OR (Depressive Symptoms):ti,ab,kw OR (Emotional Depression):ti,ab,kw OR (depress*):ti,ab,kw

#3 (random*):ti,ab,kw OR (control*):ti,ab,kw

#4 (treatment):ti,ab,kw OR (intervention):ti,ab,kw OR (therapy):ti,ab,kw OR (management):ti,ab,kw

#5 (tricyclic antidepressants):ti,ab,kw OR (TCA):ti,ab,kw OR (amitriptyline):ti,ab,kw OR (nortriptyline):ti,ab,kw OR (desipramine):ti,ab,kw OR (doxepin):ti,ab,kw OR (clomipramine):ti,ab,kw OR (dopamine agonists):ti,ab,kw OR (pramipexole):ti,ab,kw OR (rotigotine):ti,ab,kw OR (pergolide):ti,ab,kw OR (memantine):ti,ab,kw OR (ropinirole):ti,ab,kw OR (piribedil):ti,ab,kw OR (selective serotonin reuptake inhibitors):ti,ab,kw OR (SSRI):ti,ab,kw OR (citalopram):ti,ab,kw OR (escitalopram):ti,ab,kw OR (fluoxetine):ti,ab,kw OR (sertraline):ti,ab,kw OR (paroxetine):ti,ab,kw OR (fluvoxamine):ti,ab,kw OR (serotonin and norepinephrine reuptake inhibitors):ti,ab,kw OR (SNRI):ti,ab,kw OR (venlafaxine):ti,ab,kw OR (desvenlafaxine):ti,ab,kw OR (duloxetine):ti,ab,kw OR (milnacipran):ti,ab,kw OR (serotonin antagonist and reuptake inhibitor):ti,ab,kw OR (trazodone):ti,ab,kw OR (nefazodone):ti,ab,kw OR (norepinephrine reuptake inhibitor):ti,ab,kw OR (reboxetine):ti,ab,kw OR (viloxazine):ti,ab,kw OR (atomoxetine):ti,ab,kw OR (noradrenergic and specific serotonergic antidepressant):ti,ab,kw OR (NaSSA):ti,ab,kw OR (mirtazapine):ti,ab,kw OR (mianserin):ti,ab,kw OR (Norepinephrine Dopamine Reuptake Inhibitor):ti,ab,kw OR (NDRI):ti,ab,kw OR (bupropion):ti,ab,kw OR (serotonin modulator and stimulator):ti,ab,kw OR (vortioxetine):ti,ab,kw OR (monoamine oxidase inhibitors):ti,ab,kw OR (rasagiline):ti,ab,kw OR (selegiline):ti,ab,kw OR (moclobemide):ti,ab,kw OR (atypical antipsychotic drugs):ti,ab,kw OR (quetiapine):ti,ab,kw OR (olanzapine):ti,ab,kw OR (tianeptine):ti,ab,kw OR (agomelatine):ti,ab,kw OR (esketamine):ti,ab,kw

#6 #1 AND #2 AND #3 AND (#4 OR #5)

**Scopus**

( TITLE-ABS-KEY ( "Parkinson Disease" OR "Idiopathic Parkinson's Disease" OR "Lewy Body Parkinson's Disease" OR "Paralysis Agitans" OR parkinson* ) AND TITLE-ABS-KEY ( "Depression" OR "Depressive Symptoms" OR "Emotional Depression" OR depress* ) AND TITLE-ABS-KEY ( random* OR control* ) AND ( TITLE-ABS-KEY ( treatment OR intervention OR therapy OR management ) OR TITLE-ABS-KEY ( "tricyclic antidepressants" OR "TCA" OR amitriptyline OR nortriptyline OR desipramine OR doxepin OR clomipramine OR "dopamine agonists" OR pramipexole OR rotigotine OR pergolide OR memantine OR ropinirole OR piribedil OR "selective serotonin reuptake inhibitors" OR "SSRI" OR citalopram OR escitalopram OR fluoxetine OR sertraline OR paroxetine OR fluvoxamine OR "serotonin and norepinephrine reuptake inhibitors" OR "SNRI" OR venlafaxine OR desvenlafaxine OR duloxetine OR milnacipran OR "serotonin antagonist and reuptake inhibitor" OR trazodone OR nefazodone OR "norepinephrine reuptake inhibitor" OR reboxetine OR viloxazine OR atomoxetine OR "noradrenergic and specific serotonergic antidepressant" OR "NaSSA" OR mirtazapine OR mianserin OR "Norepinephrine Dopamine Reuptake Inhibitor" OR "NDRI" OR bupropion OR "serotonin modulator and stimulator" OR vortioxetine OR "monoamine oxidase inhibitors" OR rasagiline OR selegiline OR moclobemide OR "atypical antipsychotic drugs" OR quetiapine OR olanzapine OR tianeptine OR agomelatine OR esketamine ) ) )

**Web of Science**

#1 TS=("Parkinson Disease" OR "Idiopathic Parkinson's Disease" OR "Lewy Body Parkinson's Disease" OR "Paralysis Agitans" OR parkinson*)

#2 TS=("Depression" OR "Depressive Symptoms" OR "Emotional Depression" OR depress*)

#3 TS=(random* OR control*)

#4 TS=(treatment OR intervention OR therapy OR management)

#5 TS=("tricyclic antidepressants" OR "TCA" OR amitriptyline OR nortriptyline OR desipramine OR doxepin OR clomipramine OR "dopamine agonists" OR pramipexole OR rotigotine OR pergolide OR memantine OR ropinirole OR piribedil OR "selective serotonin reuptake inhibitors" OR "SSRI" OR citalopram OR escitalopram OR fluoxetine OR sertraline OR paroxetine OR fluvoxamine OR "serotonin and norepinephrine reuptake inhibitors" OR "SNRI" OR venlafaxine OR desvenlafaxine OR duloxetine OR milnacipran OR "serotonin antagonist and reuptake inhibitor" OR trazodone OR nefazodone OR "norepinephrine reuptake inhibitor" OR reboxetine OR viloxazine OR atomoxetine OR "noradrenergic and specific serotonergic antidepressant" OR "NaSSA" OR mirtazapine OR mianserin OR "Norepinephrine Dopamine Reuptake Inhibitor" OR "NDRI" OR bupropion OR "serotonin modulator and stimulator" OR vortioxetine OR "monoamine oxidase inhibitors" OR rasagiline OR selegiline OR moclobemide OR "atypical antipsychotic drugs" OR quetiapine OR olanzapine OR tianeptine OR agomelatine OR esketamine)

#6 #1 AND #2 AND #3 AND (#4 OR #5)

**Table S1: Search Strategy.**

| **Study/Year** | **Subjects**  **(intervention/control)** | **Age Mean ± SD/Mean**  **(range/median)** | **Dosage** | **Intervention/Drug class** | **Treatment duration** | **Outcome** | **Duration of PD(years)** |
| --- | --- | --- | --- | --- | --- | --- | --- |
| A. Avila et al 2003^10^ | 9  7 | NA  NA | 50-500mg  10-50mg | Nefazodone  Fluoxetine | 4 weeks | BDI | NA  NA |
|  |  |  |  |  |  |  |  |
| H. Allain. et al 1991^11^ | 48  45 | 64.5±8.5  65.4±10.1 | 10mg/day | Selegiline  Placebo | 3 months | HDRS | NA  NA |
|  |  |  |  |  |  |  |  |
| A. Makkos et al 2016^12^ | 23  21 | 67 (60-73)  66 (62-70) | 5HZ | M1-rTMS  Sham | 10 days | BDI  HDRS | 6(3-10)  5(3-9) |
|  |  |  |  |  |  |  |  |
| Carlo Cattaneo et al 2017^13^ | 180  175 | NA  NA | 100mg/day | Safinamide  Placebo | 24 months | HAMD | NA  NA |
|  |  |  |  |  |  |  |  |
| David Devos et al 2008^14^ | 15  17  16 | 57 (56–64)  63 (57–65)  65 (56–68) | 25-75mg/day  20-28mg/day | Citalopram  Desipramine  Placebo | 30 days | MADRS | 7(4–10)  9(6–13)  8(4–10) |
|  |  |  |  |  |  |  |  |
|  |  |  |  |  |  |  |  |
| E. M. Khedr et al 2024^15^ | 16  8 | 61.8±3.5  60.2±1.6 | 20HZ | **Parietal Cortex-**rTMS  Sham | 10 days | BDI | NA  NA |
|  |  |  |  |  |  |  |  |
|  |  |  |  |  |  |  |  |
| E. Pál et al 2010^16^ | 12  10 | 68.5(59.5-70.0)  67.5(57.0-72.0) | 5HZ | DLPFC-rTMS  Sham | 10 days | BDI  MADRS | 6.0(3.0-9.5)  6.5(3.8-10.5) |
|  |  |  |  |  |  |  |  |
| E. Ziaei et al 2022^17^ | 20  21 | 64±13  64±13 | 0.7-1.4mg/g  10-20mg/day | Pramipexole  Citalopram | 7 weeks | BDI | 2(2)  2(2) |
|  |  |  |  |  |  |  |  |
| I. H. Richard et al 2012^18^ | 34  30  33 | 65.2±9.8  62.5±11.4  62.7±11.0 | 10mg/day  37.5mg/day | Paroxetine  Venlafaxine  Placebo | 12 weeks | HAMD | 5.2±5.9  4.7±3.7  4.9±3.6 |
|  |  |  |  |  |  |  |  |
| Jing, Chen et al 2022^19^ | 29  31  29 | 64.34±9.68  64.35±8.93  64.17±8.37 | 10mg/day  0.375-1.5mg/day  5Hz | Escitalopram  Pramipexole  DLPFC-rTMS | 4 weeks | HAMD  PHQ-9 | 3.59±3.70  3.71±2.47  4.14±2.47 |
|  |  |  |  |  |  |  |  |
|  |  |  |  |  |  |  |  |
| I.Rektorová et al 2003^20^ | 18  16 | 59.7±7.7  63.5±7.5 | 1.5-4.5mg/day  1.5-4.5mg/day | Pramipexole  Pergolide | 8 months | SDS | 4.6(0-31)  2.6(0-9) |
| L. I. Aftanas, et al 2021^21^ | 23  23 | 63.7±8.8  62.9±7.1 | 10Hz | M1+DLPFC-rTMS  Sham | 3 weeks | HDRS-17  Depression on DASS-21  BDI-II | 7.0±4.0  5.6±4.0 |
|  |  |  |  |  |  |  |  |
|  |  |  |  |  |  |  |  |
| M. Meloni et al 2020^22^ | 23  23 | 67.6±6.6 | 50mg | 5-hydroxytryptophan  Placebo | 12 weeks | HDRS | 9.7±5.7 |
|  |  |  |  |  |  |  |  |
|  |  |  |  |  |  |  |  |
| M. Pomponi, G et al 2014^23^ | 12  12 | 64.0±4.9  64.0±9.8 | 150-400mg  150-400mg | DHA  Placebo | 6 months | HAMD | 4.6±2.7  5.0±2.4 |
|  |  |  |  |  |  |  |  |
| M. Serrano et al 2002^24^ | 37  40 | 68,3±4.2  68,1±4,9 | 20-40mg/day  25-75mg/day | Fluoxetine  Amitriptyline | 12 months | HAMD | 7±0.8  6.9±0.9 |
|  |  |  |  |  |  |  |  |
| Menza M et al 2009^25^ | 17  18  17 | 62.9±9.3  62.8±8.1  61.4±9.2 | 25-75mg  12.5-37.5mg | Nortriptyline  Paroxetine  Placebo | 8 weeks | HAMD | 6.1±7.5  8.6±6.1  4.2±7.9 |
|  |  |  |  |  |  |  |  |
| O. Rascol et al 2012^26^ | 140  133 | 62.4±8.9  62.1±9.3 | 12-24mg/day  12-24mg/day | Pardoprunox  Placebo | 7 weeks | HADS-Depression | 6.7±4.4  6.6±4.7 |
|  |  |  |  |  |  |  |  |
|  |  |  |  |  |  |  |  |
| P. Barone et al 2006^27^ | 33  34 | 64.8±8.3  68.1±6.5 | 1.5-4.5mg/day  50mg/day | Pramipexole  Sertraline | 12 weeks | HAMD  SDS | NA  NA |
|  |  |  |  |  |  |  |  |
| P. S. Boggio et al 2005^28^ | 13  12 | 28.8±3.1  35.3±5.5 | 15HZ  20mg | DLPFC-rTMS  Fluoxetine | 8 weeks | BDI  HAMD | NA  NA |
|  |  |  |  |  |  |  |  |
| P.Barone et et al 2010^29^ | 139  148 | 67.4±9.0  66.6±9.9 | 2.18mg/day | Pramipexole  Placebo | 12 weeks | BDI | 4.0±4.5  4.0±3.9 |
|  |  |  |  |  |  |  |  |
| R. Daneshvar Kakhki et al 2020^30^ | 25  26 | 66.4±8.2  66.3±9.3 | 10mg/day | Melatonin  Placebo | 12 weeks | BDI | 5.7±1.9  5.5±2.1 |
|  |  |  |  |  |  |  |  |
| R. Pahwa et al 2007^31^ | 202  191 | 66.3±99.2  66.0±99.7 | 18.8mg/day | Ropinirole  Placebo | 24 weeks | BDI | 8.6±4.8  8.6±5.2 |
|  |  |  |  |  |  |  |  |
| S. Jiang et al 2023^32^ | 27  28 | 62.7±12.9  64.3±8.9 | 10HZ | rTMS-DLPFC  Placebo | 10 days | HAMD | 7(3,10.2)  3(2,7) |
|  |  |  |  |  |  |  |  |
| S. Mehrabani et al 2023^33^ | 40  40 | 68.2±7.7  69.1±8.2 | 5 g/day | Synbiotic supplementation  Placebo | 12weeks | BDI | 5.72±2.86  5.28±1.76 |
|  |  |  |  |  |  |  |  |
| S. Thobois et al 2013^34^ | 19  18 | 58.6±6.5  55.6±8 | 300mg/day | Piribedil  Placebo | 12 weeks | BDI  HAMD | 12±3.5  11.1±2.6 |
|  |  |  |  |  |  |  |  |
| W. Koller et al 1993^35^ | 202  202  199 | NA  NA  NA | 2000IU  10mg | Tocopherol  Deprenyl  Placebo | 3 months | HAMD | NA  NA  NA |
|  |  |  |  |  |  |  |  |
|  |  |  |  |  |  |  |  |
|  |  |  |  |  |  |  |  |
|  |  |  |  |  |  |  |  |
| W. Song et al 2024^36^ | 22  22 | 67.4±7.0  70.5±6.8 | 10HZ | M1-rTMS  Placebo | 10days | HAMD | 6.2±1.6  6.8±2.0 |
|  |  |  |  |  |  |  |  |
|  |  |  |  |  |  |  |  |
| X. Zhu et al 2025^37^ | 30  30 | NA  NA | 100U | Botulinum Toxin A  placebo | 12 weeks | CMI-depression | NA  NA |
|  |  |  |  |  |  |  |  |
| Z. Zhang et al 2025^38^ | 20  20 | 69.2±5.8  71.4±6.0 | 10HZ | M1+SMA rTMS  M1 rTMS  SMA rTMS  Sham | 10 days | HAMD | 7.2±3.3  7.8±2.6 |
|  |  |  |  |  |  |  |  |
| Zhenxin Zhang et al 2013^39^ | 175  170 | 64.1±9.0  63.6±10.5 | 11.4mg/day | Ropinirole  Placebo | 24 weeks | HAMD | 7.7±5.0  8.0±4.0 |
|  |  |  |  |  |  |  |  |
|  |  |  |  |  |  |  |  |
| P. Barone et al 2015^40^ | 58  65 | 66.0 ± 8.74  66.1±8.35 | 1mg/day | Rasagiline  Placebo | 4 weeks | BDI | 3.7±3.17  4.8±3.78 |
|  |  |  |  |  |  |  |  |
| D. H. Benninger et al 2010^41^ | 13  12 | 63.6±9.0  64.2±8.8 | 2mA 20min | Transcranial Direct Current Stimulation (tDCS)  Sham | 3 months | BDI | 10.6±7.1  9.1±3.3 |
| M. Bryś et al 2016^42^ | 14  12  15 | 59.6±12.6  64.6±12.3  64.0±7.4 | 10Hz | M1-rTMS  DLPFC-rTMS  Sham | 10 days | HAMD | 8.4±5.2  7.7±4.2  4.5±2.2 |
|  |  |  |  |  |  |  |  |
| A. Castrioto et al 2020^43^ | 26  22 | 57.1±6.5  60.9±8.4 | 8mg/day | Rotigotine  Placebo | 6 months | BDI | 2.2±1.4  2±1.5 |
| Y. Cheng et al 2023^44^ | 27  27 | 60.5±8.7  62.6±8.4 | NA | Fecal Microbiota Transplantation (FMT)  Placebo | 6 weeks | PHQ-9 | 6.7±4  5.9±4.4 |
|  |  |  |  |  |  |  |  |
| S. J. Chung et al 2016^45^ | 184  196 | 65.6±8.9  64.9±8.2 | 8mg/day | Rotigotine  Placebo | 8 weeks | HAMD | 2.99±3.26  2.51±2.90 |
| A. J. Espay et al 2011^46^ | 12  11 | 67.6±9.6 | 80mg/day | Methylphenidate  Placebo | 6 months | HADRS | 10.6±11 |
|  |  |  |  |  |  |  |  |
| H. Hanaĝasi et al 2011^47^ | 23  25 | 65.17±9.5  67.56±10.13 | 1mg/day | **Rasagiline**  **Placebo** | 12 weeks | GDS | 4.09±2.54  3.96±2.26 |
| A. Kanjanarangsichai et al 2022^48^ | 20  20 | NA | 15mg/day | Cannabidiol-enriched Cannabis  Placebo | 8 weeks | HADS | NA |
|  |  |  |  |  |  |  |  |
| D. Athauda et al 2017^49^ | 31  29 | 61.6±8.2  57.8±8.0 | 2mg/week | Exenatide  Placebo | 60 weeks | MADRS | 5.7±7.9  5.6±7.7 |
| W.G. Ondo et al 2005^50^ | 20  20 | 64.4±10.4  65.1±12.3 | 200mg/day | Modafinil  Placebo | 4 weeks | HAMA | 6.5±5.5  7.0±4.6 |
|  |  |  |  |  |  |  |  |
| Y .Shirota et al2013^51^ | 36  36 | 68.8±7.6  65.7±8.5 | 1 Hz | SMA rTMS  Sham | 8 weeks | HAMD | 8.5±7.3  7.6±4.4 |
| A.Siderowf et al  2002^52^ | 134  138 | 61.6±10.3  60.5±10.8 | 1mg/day | Rasagiline  Placebo | 26 weeks | BDI | 0.92±1.24  0.94±1.10 |
|  |  |  |  |  |  |  |  |
| K.L.Tsang et al 2000^53^ | 20  20 | 66.9±5.5  65.3±4.9 | 0.625mg/day | Estrogen  Placebo | 8 weeks | HAMD | 14.8±4.5  13.5±4.4 |
|  |  |  |  |  |  |  |  |
| L.Wermuth et al 1998^54^ | 18  19 | 65.9(44-79)  63.4(47-79) | 10mg/day | Citalopram  Placebo | 52 weeks | HAMD | NA |
|  |  |  |  |  |  |  |  |
| W. Mitarnun et al 2025^55^ | 30  30 | 59.8±7.59  58.7±11.15 | NA | Cannabidiol  Placebo | 12 weeks | HADS-D | 4.9±3.46  4.2±2.94 |
| L. Magistrelli et al 2024^56^ | 19  19 | 67.32±7.03  69.40±7.51 | NA | Probiotics  Placebo | 12 weeks | BDI | 3.79±1.18  4.05±0.94 |
|  |  |  |  |  |  |  |  |
| R. A. Hauser et al 2016^57^ | 41  40 | 70.2±8.0  69.0±11.7 | 8-16mg/day | Rotigotine  Placebo | 12 weeks | BDI | 4.8±4.3  3.7±3.7 |
| Werneck et al 2009^58^ | 8  12 | 62.3(45-75) | 100mg/day | Trazodone  Placebo | 5 months | HAMD | NA |
|  |  |  |  |  |  |  |  |
| Trenkwalder et al.  2011^59^ | 191  96 | 64.8±9.3  64.4±10.6 | 2-16mg/day | Rotigotine  Placebo | 10 weeks | BDI | 4.6±4.2  4.9±4.6 |
| W.G. Ondo et al  2011^60^ | 20  20 | 69.2±7.9  68.9±8.4 | 20mg/day | Memantine  Placebo | 4 weeks | MADRS | NA |

**Table S2: Characteristics of included RCTs.**

| **Study** | **Stimulating area** | **frequency** | **intensity** | **pulse number** | **treatment course** |
| --- | --- | --- | --- | --- | --- |
| A. Makkos et al 2016^12^ | Bilateral primary motor cortex, M1 | 5 Hz | 90% resting motor threshold (90% RMT) | 600 **pulses/day**  **(**right 300 + left 300） | 10 days |
| E. M. Khedr et al 2024^15^ | Parietal cortex | 20 Hz | 80% RMT | **4000 pulses/session**（P4 2000 + P3 2000） | 10 days |
| E. Pál et al 2010^16^ | Left dorsolateral prefrontal cortex, Left DLPFC | 5 Hz | 90% RMT | 600 pulses/session | 10 days |
| Jing, Chen et al 2022^19^ | left dorsolateral prefrontal region | 5 Hz | NA | **NA** | 30 days |
| L. I. Aftanas, et al 2021^21^ | M1 and left DLPFC | 10 Hz | M1 100%MT Left DLPFC 110% MT | M1 4000 Left DLPFC 3000 | 20 days |
| P. S. Boggio et al 2005^28^ | Left DLPFC | 15 Hz | 110% MT | **3000** | 2 weeks |
| S. Jiang et al 2023^32^ | Left DLPFC | 10 Hz | 100%RMT | 1200 | 10 days |
| W. Song et al 2024^36^ | Bilateral M1 | 10 Hz | 90% RMT | **1000** | 10 days |
| Z. Zhang et al 2025^38^ | M1  SMA | 10 Hz | 100% RMT | 1200 | 10 days |
| M. Bryś et al 2016^42^ | M1  Left DLPFC  Right DLPFC | 10 Hz | NA | **2000**  **1000**  **1000** | 10 days |
| Y .Shirota et al2013^51^ | SMA | 1 Hz | 110% RMT | 1000 | 8 weeks |

**Table S3: Characteristics of included rTMS RCTs.**

**Fig S1: Risk of bias assessment tool 2 of RCTs.**

**Fig S2：Pairwise meta-analysis comparing the efficacy of DLPFC rTMS for PD patients with depression.**

**Fig S3：Sensitivity analysis of DLPFC rTMS efficacy in PD patients with depression.**

**Fig S4：Pairwise meta-analysis comparing the efficacy of M1 rTMS for PD patients with depression.**

**Fig S5：Sensitivity analysis of M1 rTMS efficacy in PD patients with depression.**

**Fig S6：Pairwise meta-analysis comparing the efficacy of rotigotine for PD patients with depression.**

**Fig S7：Pairwise Meta-Analysis Comparing the Safety of rTMS for PD patients with depression.**

**Fig S8：Pairwise meta-analysis comparing the safety of rotigotine for PD patients with depression.**

**Fig S9：Pairwise meta-analysis comparing the withdrawal rate of rotigotine for PD patients with depression.**

**Fig S10: Forest plot of treatment efficacy in NMA.**

**Fig S11: Forest plot of adverse reaction incidence in NMA.**

**Fig S12: Forest plot of adverse event-related withdrawal in NMA.**

**Fig. S13: Comparison-adjusted funnel plot for all treatments network.**
